# Accessibility of Essential Medicines for Children in Sichuan Province of China: A Cross-Sectional Study

**DOI:** 10.3389/fphar.2022.828152

**Published:** 2022-02-16

**Authors:** Zhe Chen, Siyu Li, Imti Choonara, Linan Zeng, Zhi-jun Jia, Guo Cheng, Qin Yu, Lingli Zhang

**Affiliations:** ^1^ West China School of Pharmacy, Sichuan University, Chengdu, China; ^2^ Department of Pharmacy, West China Second University Hospital, Sichuan University, Chengdu, China; ^3^ Evidence-based Pharmacy Center, West China Second University Hospital, Sichuan University, Chengdu, China; ^4^ Key Laboratory of Birth Defects and Related Diseases of Women and Children, Ministry of Education, Chengdu, China; ^5^ West China School of Medicine, Sichuan University, Chengdu, China; ^6^ Academic Division of Child Health, Derbyshire Children’s Hospital, University of Nottingham, Derby, United Kingdom; ^7^ Department of Pediatrics, West China Second University Hospital, Sichuan University, Chengdu, China; ^8^ Laboratory of Molecular Translational Medicine, Center for Translational Medicine, Sichuan University, Chengdu, China; ^9^ National Drug Clinical Trial Institute, West China Second University Hospital, Sichuan University, Chengdu, China

**Keywords:** children, medicine, accessibility, availability, price, affordability, cross-sectional study

## Abstract

**Background:** Essential medicines for children are those medicines that satisfy the priority health care needs of children. Access to essential medicines for children is a big challenge, particularly in low- and middle-income countries. Our study aimed to assess the accessibility of essential medicines for children in public sector in Sichuan Province of China, based on availability, affordability, and price.

**Methods:** We adopted the modified World Health Organization/Health Action International (WHO/HAI) standardization methodology to measure the availability, affordability, and prices of 30 essential medicines for children in 20 public hospitals in nine regions of the Sichuan Province, China. Availability was expressed as the percentage of public medicine outlets that stocked surveyed medicines on the day of data collection, and prices were expressed as median price ratio (MPR). Affordability was assessed as the number of Sichuan Province’s daily wages required for the lowest-paid government unskilled worker (11.03 USD per day) to purchase one standard treatment of an acute disease or treatment for chronic disease for a month.

**Results:** The mean availability of originator brands (OBs) and lowest priced generics (LPGs) were 9.7 and 46.5% in public sector. MPRs of only 3 OBs could be calculated, ranging from 0.55 to 13.37. MPRs of 18 LPGs ranged from 0.07 to 25.05. Among them, 2 OBs and 11 LPGs were priced at more than 1.5 times their international reference prices (IRPs) in public sector, most of which were injections. Except for cefazolin injection and ceftriaxone injection, most LPGs were affordable for the treatment of childhood diseases in public sector, as they each cost one or less than the daily wage for the lowest-paid unskilled government worker.

**Conclusions:** Although the availability of LPGs for children was higher than OBs in public sector, the availability of children’s essential medicines was low in surveyed public sector in Sichuan Province, which was similar to previous studies in other provinces of China. The price of most medicines surveyed was higher than their IRPs in surveyed public sector, especially for some injections. The affordability of most surveyed LPGs was reasonable in surveyed public sector, except for ceftriaxone injection and cefazolin injection.

## Introduction

Access to health care is a fundamental human right, and the provision of affordable, high-quality, and appropriate medicines for children is a vital part of a well-functioning health system (Zhang et al., 2017). To improve access to essential medicines for children and ensure that children’s basic medication needs can be met, the WHO has developed and revised seven editions of *Essential Medicine Lists For Children* since 2007 (World Health Organization, 2019). WHO also launched the “Make Medicines Child Size” campaign and “Better Medicines for Children” initiative in 2009 to ensure that children’s medicines were in appropriate formulations, safe and effective (World Health Organization, 2017; Finney, 2019).

Nowadays, a systematic review has identified 18 multicentre studies evaluating the accessibility of essential medicines for children (Chen et al., 2021). The majority of studies used the WHO/HAI standardized methodology (World Health Organization and Health Action International, 2008), which was jointly developed by WHO and HAI in 2000 for standardizing studies on the accessibility of medicines. This ensured the quality of studies, and made the study results in different regions comparable. The WHO/HAI standardized methodology estimates the accessibility of medicines by investigating medicine availability, price, and affordability. The results have shown that the availability of essential medicines for children is generally low in low-income and middle-income countries, the median price ratios of originator brands was higher than that of lowest-priced generics, and the most lowest-priced generics had better affordability (Chen et al., 2021).

In China, there is no essential medicine list for children, and the accessibility of essential medicines for children is still low and urgently needs to be improved (Wang et al., 2017). Moreover, the scope of medical insurance coverage for children is limited. Generally, hospitalization expenses for children can be reimbursed. But there are differences in the proportion of medical insurance payments in different levels of hospitals. Only a portion of medical expenses and the drugs are included in the *Basic Medical Insurance, Work Injury Insurance and Maternity Insurance Drug List.* Similarly, outpatient expenses for children at designated medical facilities are also reimbursed, but there is a limit to the total reimbursement of outpatient expenses.

At present, there is no study on the accessibility of essential medicines for children in Sichuan Province, China. The relevant evaluation data needs to be enriched. Therefore, the WHO/HAI standardized methodology is used to investigate the availability, price, and affordability of essential medicines for children in public sector in Sichuan Province, China.

## Materials and Methods

### Study Design

This study was a cross-sectional study on the accessibility of children’s essential medicines conducted by using the WHO/HAI standardized methodology in Sichuan Province, China (World Health Organization and Health Action International, 2008).

### Setting

**FIGURE 1:**
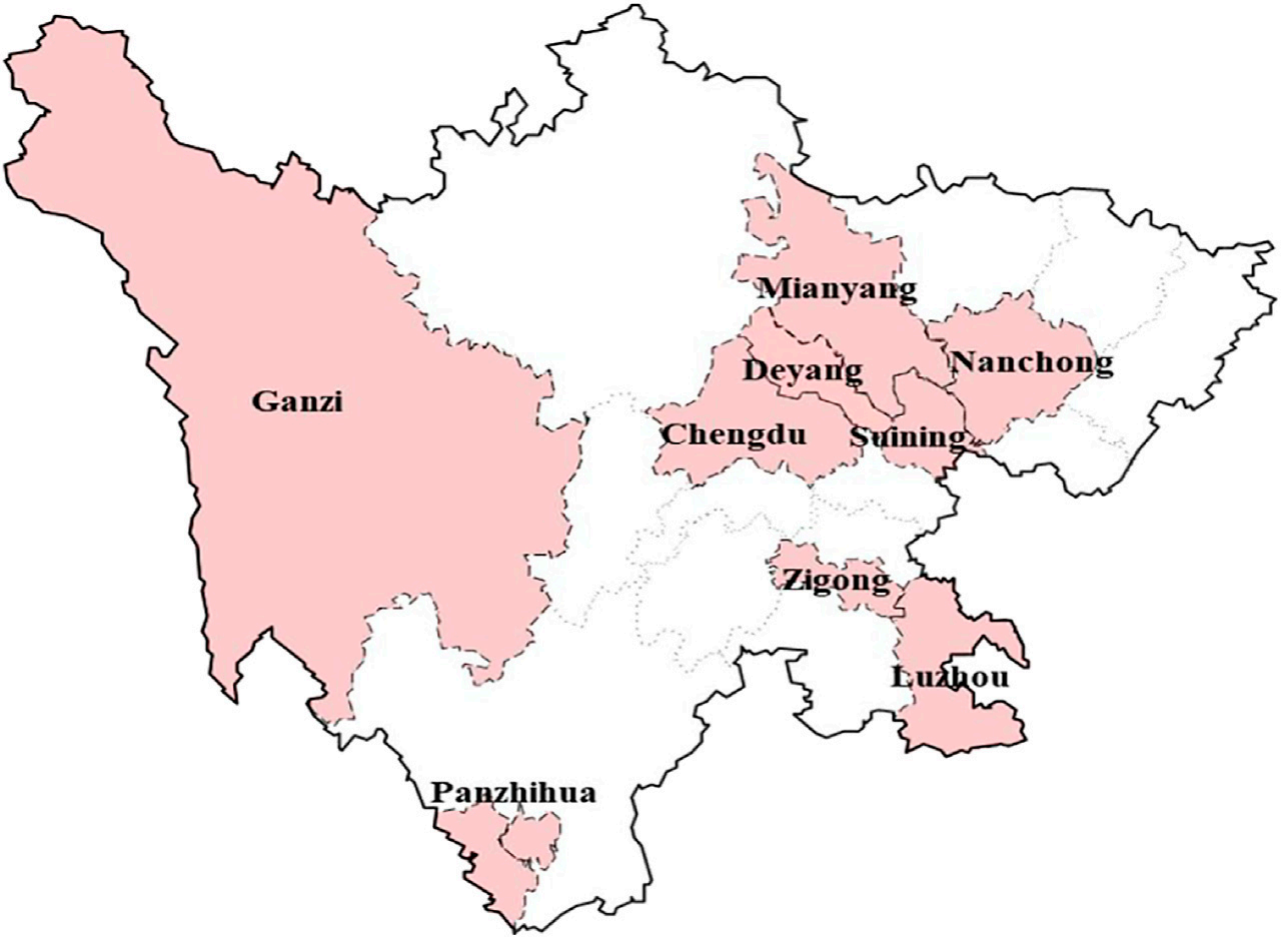
Cities of Sichuan province included in the study.

Sichuan Province is located in southwestern China, with 21 cities and a population of 91 million. Chengdu, the capital city of Sichuan Province, was chosen as the major urban center. Considering geographical position and level of economic development (Gross Domestic Product), 9 representative cities were selected as survey areas for data collection by stratified sampling: Chengdu, Mianyang, Deyang, Nanchong, Luzhou, Zigong, Suining, Panzhihua, and Ganzi. The selected cities can be reached within 1 day of travel from the capital city (Figure 1).

### Participants

We designed a sampling frame for the public sector facilities. Convenience sampling was used in our study, where subjects were selected by their convenient accessibility to the researchers. Considering the hospital level and distribution of specialist children’s hospitals, we finally selected 10 specialist children’s hospitals in Chengdu, Mianyang, Luzhou, and 10 general hospitals with pediatrics outpatient departments in Chengdu, Mianyang, Deyang, Nanchong, Luzhou, Zigong, Suining, Panzhihua, and Ganzi. Because not each of the cities studied has a specialist children’s hospital.

According to the requirements of the WHO/HAI guide book, core and supplementary medicines need to be determined in the survey following local medical burden, treatment resource, and prognosis management (World Health Organization and Health Action International, 2008). A total of 30 medicines were surveyed, all of which were registered in China (National Health Commission of China, 2018) (Table 1). Among them, 22 medicines were identified as core medicines referred from “2019 World Health Organization Model List of Essential Medicines for Children” and “2018 National Essential Medicine list of China” (National Health Commission of China, 2018; World Health Organization, 2019). These core medicines all have international reference prices (IRPs). Eight medicines were identified as supplementary medicines from the “2018 National Essential Medicine list of China.” The medicines were selected based on the local children’s disease burden and needs, literature reviews, and were assessed by pediatric clinical pharmacists.

**TABLE 1:** List of medicines assessed in the study.

Medicine	Strength	Pharmaceutical form
Core medicines		
Amoxicillin	250 mg	Tablet/Capsule
Ampicillin	1 g	Injection
Fluconazole	0.2 g/100 ml	Injection
Cefazolin	1 g	Injection
Ceftriaxone	250 mg	Injection
Phenobarbital	30 mg	Tablet
Phenytoin sodium	50 mg	Tablet
Midazolam	5 mg/ml	Injection
Diazepam	10 mg/2 ml	Injection
Isoniazid	100 mg	Tablet
Salbutamol^*^	100 mcg/dose	Inhaler
Adrenaline	1 mg/ml	Injection
Loratadine	1 mg/ml	Oral liquid
Hydrochlorothiazide	25 mg	Tablet
Furosemide	20 mg/2 ml	Injection
Metronidazole	0.5 g/100 ml	Injection
Piperacillin + Tazobactam	4.5 g	Injection
Rifampin	150 mg	Capsule
Acyclovir	0.2 g	Tablet
Omeprazole	10 mg	Tablet
Carbamazepine^*^	100 mg	Tablet
Levothyroxine	50 μg	Tablet
**Supplementary medicines**		
Oral rehydration salts	5.125 g	Powder
Albendazole	200 mg	Tablet
Amoxicillin + Clavulanic Acid^*^	200 mg: 28.5 mg	Suspension
Azithromycin^*^	100 mg	Suspension
Digoxin	0.5 mg/2 ml	Injection
Prednisone	5 mg	Tablet
Ibuprofen	2 g/100 ml	Suspension
Vitamin K1	10 mg/ml	Injection

The core medicines were both in the “2019 World Health Organization Model List of Essential Medicines for Children” and “2018 National Essential Medicine list of China.” The supplementary medicines were only in the “2018 National Essential Medicine list of China.”

* The medicine did not have international reference prices.

For each medicine, price and availability data were collected for two products: the originator brand (OB) and the lowest price generic (LPG) equivalent. The OB product was defined as a product marketed by the originator pharmaceutical company. The LPG was defined as the same efficacy product sold under the generic name with the lowest unit price at each public medicine outlet at the time of data collection in the survey.

### Variables

The WHO/HAI methodology recommended assessing the accessibility of medicines by three indicators, including availability, price and affordability.

Availability of medicines was expressed as the percentage (%) of pharmacies in which the individual medicines were available on the day of the survey.

Price was expressed by the median price ratios (MPR). The MPR was the ratio of the median medicine unit (each tablet, capsule, and milliliter, etc.) price to IRP (World Health Organization and Health Action International, 2008). The IRPs were the medians of recent procurement prices offered for medicines by not-for-profit suppliers. For instance, MPR was 2.0 meant that the local medicine price was 2 times the IRP. If the retail price of medicines in public sectors did not exceed 1.5 times the IRP, namely MPR ≤ 1.50, which was considered reasonable (Wang et al., 2017; Hailu and Mohammed, 2020; Abrha et al., 2018).

Affordability was assessed by comparing the daily wage of lowest-paid unskilled government workers and medicines expenses in the standard treatment of childhood diseases (a full course of therapy for acute diseases or the cost of a 30-days supply of medicines for chronic diseases). Medicines were considered affordable if the medicines cost less than the daily wage (Sado and Sufa, 2016). According to child disease burden from WHO (World Health Organization, 2019), the sixth national health service survey on child healthcare in China, and pediatric experts’ opinions, six common conditions in childhood were chosen to assess the affordability, including bacterial upper respiratory tract infection, urinary tract infection, community-acquired pneumonia, epilepsy, asthma, fever. The medicines recommended for these conditions were listed in Table 2 (Working Group on Revision of Guidelines for the Clinical Application of Antimicrobial Drugs, 2015; Respiratory Group of Pediatrics Branch of Chinese Medical Association, 2016; Luo et al.,2016). The dosage and duration of medicine treatment was the standard treatment recommended by (Working Group on Revision of Guidelines for the Clinical Application of Antimicrobial Drugs, 2015), guideline (Luo et al., 2016) or *China National Formulary (Children’s Edition)* (China National Formulary Editorial Board, 2013). Sichuan Province’s daily wage of the lowest-paid government unskilled employee was 71.26 CNY (approximately 11.03 USD in 7 May 2021) (The People’s Government of Sichuan Province, 2018). Besides, we assumed the child was 5 years old with a weight of 20.00 kg (National Health Commission of the People’s Republic of China, 2016).

**TABLE 2:** The medicines recommended for these conditions in children.

Conditions	Recommended treatment	
	First line treatment	Second line treatment
Bacterial upper respiratory tract infection	penicillin G, penicillin V, amoxicillin	the first or second-generation cephalosporins
Urinary tract infection	ampicillin, amoxicillin, the first or second or third generation cephalosporins	piperacillin/tazobactam, ampicillin/sulbactam, amoxicillin/clavulanic acid, fluoroquinolones, carbapenems
Community-acquired pneumonia	amoxicillin, amoxicillin/clavulanic acid	the first or second-generation cephalosporins
Epilepsy	sodium valproate (except for partial seizure), carbamazepine (partial seizure), levetiracetam, topiramate, lamotrigine	—
Asthma	short-acting Beta2 agonist, short-acting muscarinic antagonist, inhaled corticosteroids, long-acting Beta2 agonist, leukotriene receptor antagonists	—
Fever	ibuprofen, acetaminophen	—

### Data Sources

We sent a special standardized electronic data collection format to each hospital pharmacy to collect information on availability and price. To verify the standardized data collection form and ensure data accuracy and reliability, we conducted a pilot study and trained the person who filled out the data collection form. We checked the completed data collection forms and verified the data integrity as the forms were submitted. Then, two well-trained researchers independently entered the survey data into the standardized WHO/HAI Excel Workbook (World Health Organization and Health Action International, 2008) using a double-entry technique. All data on the availability and prices of medicines in public sector was collected from 1 July 2021 to 10 July 2021.

### Statistical Methods

Data were cleared and analyzed by using MS Excel Workbook provided by World Health Organization and Health Action International, 2008 part I. The median availability was calculated for OBs and LPGs for the overall basket of medicines surveyed within public sector. For medicine prices, we used the International Reference Price (IRP) in 2015 (International Drug Price Indicator Guide, 2015) published by Management Sciences for Health to calculate MPR of each medicine. MPR was only calculated when the medicine was available at a minimum of four public medicine outlets (Sun et al., 2018). The exchange rate used to calculate MPRs was 1 CNY = 0.15 USD in 7 May 2021, which was provided by the State Administration of Foreign Exchange (State Administration of Foreign Exchange, 2021).

## Results

Ten specialist children’s hospitals and 10 general hospitals participated in the survey and returned the questionnaire.

Among 30 OBs, 10 OBs were assessed for availability. Availability of 7 OBs were less than 50.0%, 3 OBs were between 50.0 and 80.0%, and no OB was found in more than 80.0% of public medicine outlets. Among 30 LPGs, 29 LPGs were assessed for availability. Availability of 15 LPGs were less than 50.0%, 5 LPGs were between 50.0 and 80.0%, and 9 LPGs were found in more than 80.0% of public medicine outlets. Neither the OB nor the LPG of digoxin injection was not found in any public medicine outlet (Table 3). The mean availabilities of OBs and LPGs were 9.7% (SD 18.9%) and 46.5% (SD 32.5%) in public sector. The median availability of all OBs and all LPGs were 0.0 and 40.0%. Though the availability of LPGs was higher than OBs, the availability of OBs and LPGs were both low. The availability of LPGs seemed to be no different in specialist hospitals and general hospitals (Table 4). No hospital had all the medicines or had none of the medicines available. The hospital having the most surveyed medicines was a general hospital and 25 medicines were available. The hospital having the least surveyed medicines was a specialist hospital and 6 medicines were available.

**TABLE 3:** Availability of individual medicines for children.

Medicine (Strength, pharmaceutical form)	Availability	
	Originator Brands (%)	Lowest Price Generics (%)
Amoxicillin (250 mg, tab/cap)	0.0	85.0
Ampicillin (1 g, inj)	0.0	50.0
Fluconazole (0.2 g/100 ml, inj)	15.0	35.0
Cefazolin (1 g, inj)	0.0	45.0
Ceftriaxone (250, inj)	5.0	10.0
Phenobarbital (30 mg, tab)	0.0	55.0
Phenytoin sodium (50 mg, tab)	0.0	15.0
Midazolam (5 mg/ml, inj)	0.0	15.0
Diazepam (10 mg/2 ml, inj)	0.0	90.0
Isoniazid (100 mg, tab)	0.0	60.0
Salbutamol (100 mcg/dose, inh)	50.0	35.0
Adrenaline (1 mg/ml, inj)	0.0	90.0
Loratadine (1 mg/ml, Oral liquid)	5.0	20.0
Hydrochlorothiazide (25 mg, tab)	0.0	85.0
Furosemide (20 mg/2 ml, inj)	0.0	95.0
Metronidazole (0.5 g/100 ml, inj)	0.0	85.0
Piperacillin + Tazobactam (4.5 g, inj)	15.0	20.0
Rifampin (150 mg, cap)	0.0	70.0
Acyclovir (0.2 g, tab)	0.0	15.0
Omeprazole (10 mg, tab)	25.0	5.0
Oral rehydration salts (5.125 g, powd)	0.0	80.0
Albendazole (200 mg, tab)	35.0	20.0
Amoxicillin + Clavulanic Acid (200 mg: 28.5 mg, susp)	0.0	20.0
Carbamazepine (100 mg, tab)	0.0	55.0
Azithromycin (100 mg, susp)	50.0	25.0
Digoxin (0.5 mg/2 ml, inj)	0.0	0.0
Prednisone (5 mg, tab)	0.0	90.0
Ibuprofen (2 g/100 ml, susp)	15.0	15.0
Vitamin K1 (10 mg/ml, inj)	0.0	95.0
Levothyroxine (50 μg, tab)	75.0	15.0

**TABLE 4:** Mean and median availability of medicines for children.

Type	The mean availability (%)			The median availability (%)		
	Specialist hospitals (*n* = 10)	General hospitals (*n* = 10)	All medicine outlets (*n* = 20)	Specialist hospitals (*n* = 10)	General hospitals (*n* = 10)	All medicine outlets (*n* = 20)
Originator Brands	8.0	13.3	9.7	0.0	0.0	0.0
Lowest Price Generics	42.3	50.7	46.5	35.0	45.0	40.0

MPRs of only 3 OBs could be calculated, ranging from 0.55 to 13.37. MPRs of 18 LPGs could be calculated, ranged from 0.07 to 25.05. Among them, 2 of 3 OBs’ MPRs and 11 of 18 LPGs’ MPRs were >1.50, namely, those medicines’ prices were considered irrational. Among LPGs, the median of MPRs for injection was 4.32 and was much higher than the tablet/capsule whose median of MPRs was 0.68. The number of injections and tablets/capsules whose MPR was >1.50 were 7 and 2, respectively, (Table 5).

**TABLE 5:** MPRs of individual medicines for children.

Medicine (Strength)	Originator Brands	Lowest Price Generics
**Tablet/Capsule**		
Amoxicillin (250 mg)	—	0.68
Phenobarbital (30 mg)	—	6.81
Phenytoin sodium (5 mg)	—	—
Isoniazid (100 mg)	—	0.41
Hydrochlorothiazide (25 mg)	—	1.11
Rifampin (150 mg)	—	0.25
Acyclovir (0.2 g)	—	—
Albendazole (200 mg)	13.37	8.80
Carbamazepine (100 mg)	—	—
Prednisone (5 mg)	—	0.61
Levothyroxine (50 μg)	0.55	—
Omeprazole (10 mg)	6.24	—
**Injection**		
Ampicillin (1 g)	—	17.26
Midazolam (5 mg/ml)	—	—
Fluconazole (0.2 g/100 ml)	—	0.07
Cefazolin (1 g)	—	12.69
Ceftriaxone (250)	—	—
Diazepam (10 mg/2 ml)	—	25.05
Adrenaline (1 mg/ml)	—	4.24
Furosemide (20 mg/2 ml)	—	4.32
Metronidazole (0.5 g/100 ml)	—	0.95
Digoxin (0.5 mg/2 ml)	—	—
Vitamin K1 (10 mg/ml)	—	9.08
Piperacillin + Tazobactam (4.5 g)	—	1.81
**Suspension**		
Amoxicillin + Clavulanic Acid (200 mg: 28.5 mg)	—	—
Azithromycin (100 mg)	—	—
Ibuprofen (2 g/100 ml)	—	—
**Inhalation**		
Salbutamol (100 mcg/dose)	—	—
**Oral liquid**		
Loratadine (1 mg/ml)	—	8.81
**Prower**		
Oral rehydration salts (5.125 g)	—	13.73

MPR, is the ratio of median medicine unit (each tablet, capsule, and milliliter, etc.) price to international reference price.

The affordability of standard treatments for six different health conditions was calculated. Due to the low availability of OBs, we finally included seven LPGs. Amoxicillin capsule, carbamazepine tablet, salbutamol inhaler, ibuprofen suspension treatment cost less than the daily wage, which meant these medicines were affordable. Amoxicillin/clavulanic acid suspension treatment cost for community-acquired pneumonia was 1.41, which was slightly over the daily wage. But the ceftriaxone injection treatment cost far more than the daily wage and was unaffordable in the treatment of urinary tract infection. The cefazolin injection treatment also cost over the daily wage and was unaffordable in the treatment of urinary tract infection and community-acquired pneumonia. Excepting ceftriaxone injection and cefazolin injection, the cost of most LPGs in public sectors was between 0.02 and 1.41 the daily wage, which indicated that most LPGs were modestly affordable in Sichuan Province(Table 6).

**TABLE 6:** Affordability of medicines for children.

Condition	Medicine (Strength, pharmaceutical form)	Lowest unit price (CNY)	Dosage, duration	Medicine expense (CNY)	The ratio of medicine expense and daily wage of lowest-paid unskilled government workers^a^
Bacterial upper respiratory tract infection	Amoxicillin (250 mg, tab/cap)	0.07	250 mg, 3 times/day, 10 day	2.1	0.03
Urinary tract infection	Amoxicillin (250 mg, tab/cap)	0.07	250 mg, 3 times/day, 14 days	2.94	0.04
	Cefazolin (1 g, inj)	39.50	Maximum 100 mg/(kg* day), 14 days	1,106.00	15.52
	Ceftriaxone (250 mg, inj)	29.80	Maximum 80 mg/(kg*day), 14 days	2,670.08	37.50
Community-acquired pneumonia	Amoxicillin (250 mg, tab/cap)	0.07	250 mg, 3 times/day, 14 days	2.94	0.04
	Amoxicillin + Clavulanic Acid (200 mg: 28.5 mg, susp)	3.59	200 mg: 28.5 mg, 2 times/day, 14 days	100.52	1.41
	Cefazolin (1 g, inj)	39.50	Maximum100 mg/(kg*day), 14 days	1,106.00	15.52
Epilepsy	Carbamazepine (100 mg, tab)	0.19	Maximum 400 mg/day, 30 days	22.80	0.32
Asthma	Salbutamol (0.1 mg/dose, inh)	0.14	Maximum 0.2 mg, 4 times/day, 30 days	33.60	0.47
Fever	Ibuprofen (2 g/100 ml, Susp)	0.4	Maximum 5 ml, 4 times/day, 3 days	24.00	0.34

* Daily wage of the lowest-paid government was 71.26 CNY, in Sichuan Province, China.

## Discussion

This study assessed the availability, price, and affordability of essential medicines for children by using the standardized WHO/HAI methodology in public sector in the Sichuan Province of China. The mean availability of originator brands (OBs) and lowest priced generics (LPGs) were 9.7 and 46.5% in public sector. MPRs of only 3 OBs could be calculated, ranging from 0.55 to 13.37. MPRs of 18 LPGs ranged from 0.07 to 25.05. Among them, 2 OBs and 11 LPGs were priced at more than 1.5 times their IRP in public sector, most of which were injections. Except for cefazolin injection and ceftriaxone injection, most LPGs were affordable for the treatment of childhood diseases in public sector, as they each cost one or less than the daily wage for the lowest-paid unskilled government worker.

There were several limitations in this study. The availability of medicine was collected and assessed on the survey day, which could not truly reflect the monthly or annual mean medicine supply. Moreover, this study was conducted only in public sector, not in private sector. The results might underestimate the accessibility of essential medicines for children, especially for OBs. The previous studies showed the availability in the private sector was higher than that in public sector (Wang et al., 2017; Sun et al., 2018). And with the continuous deepening of medical system reform and the development of Internet technology in China, the “Internet + medical” model has emerged (NHCPRC and NATCM, 2018; NHCPRC, 2020). Sichuan Province actively has supported and promoted the construction of prescription circulation platforms to provide patients with the entire chain of Internet pharmacy services, including drug delivery (HCSP and SPATCM, 2020; PAGP, 2020). Patients with the electronic prescription can independently purchase medicines in hospitals, online private pharmacies, or the third-party agency delivery services. For patients with stable chronic diseases, they may be inclined to obtain medicines at nearby physical or online private pharmacies (Wang et al., 2020). This “Internet + medical” model could increase availability of medicine in private sector. In addition, for the convenience of calculations, we selected 5-year-old children and their average weight to estimate the affordability of essential medicines for children. Thus, the medicine affordability might be overestimated, especially for older and heavier children, because they might need to take larger doses of medicines during treatment.

The previous similar studies were conducted in other provinces of China (Wang et al., 2017; Sun et al., 2018; Wei et al., 2019; Li et al., 2018; Dai, et al., 2020; Wang et al., 2020) and other low-income and middle-income countries (Anson et al., 2012; Swain et al., 2015; Sado and Sufa, 2016), which had similar results. The results showed that the availability of essential medicines for children in public sector was low. The median availability of existing lowest price generic medicines and their original products was less than 50% in public sector. Although the availability of LPGs was higher than OBs, the availability of LPGs also was low, the median was 40%. Compared with the studies conducted by Wang et al. (2017) in China’s Shaanxi Province, Sun et al. (2018) in China’s Jiangsu Province, and Wei et al. (2019) in eastern China, our findings showed lower availability of OBs and slightly higher availability of LPGs in public sector. In the survey of Jiangsu Province published in 2018, the mean availability of OBs and LPGs were 7.5 and 34.2% in the public hospitals (Sun et al., 2018).

Many studies reported high rates of off-label in different pediatric patients, which increased the risk of medicine use in children (Kimland and Odlind, 2012; Gore et al., 2017). The low availability of medicines for children and the lack of medicines with child-friendly pharmaceutical form and dose definitely deteriorated this phenomenon further. In 2018–2019, in response to the lack of standardized management of shortage medicines in China’s medical institutions, the National Health Commission of China has developed *Guidelines for the Management of Shortage Medicines in Public Medical Institutions* and *Technical Guide for the Classification, Categorization and Alternative Use of Shortage Medicines in Medical Institutions.* According to the guidelines, when medicines are not available in the public medical institutions, the following actions are needed: the pharmacists need to confirm the information of shortage medicines, including but not limited to the reasons for the medicine shortage, the main affected patient population, the procurement history and actual use of shortage medicines, estimated duration of shortage, availability of shortage medicines in other hospitals. If the medicines are available in other medical institutions, the pharmacists can initiate a purchase path. The patient can then use the medicines after purchase according to the medicine procurement process of the institution. Otherwise, if the medicines are unavailable in other medical institutions, pharmacists should classify the shortage medicines according to necessity and urgency, and carry out evidence-based selection of alternative medicines. If the alternative medicines are in the institution’s medicine supply list, it can be used directly, otherwise, the Pharmaceutical Administration and Therapeutics Committee (Group) may initiate emergency procurement according to relevant regulations of the institution, and timely notify the information of alternative medicines in the hospital.

These reasons may explain the poor availability of medicines for children. Because of low-profit margins, Chinese pharmaceutical manufacturers lack the motivation to produce children’s medicines (Jiang, 2021). Children’s medicines generally need to be made into child-friendly pharmaceutical form, such as syrups, granules, patches, and suppositories, etc. The production process of these pharmaceutical form is more complicated because these pharmaceutical form require higher medicine stability, which increases the costs and reduces profits. Although there are more than 4,000 pharmaceutical manufacturers in China, only approximately 5.0% of the pharmaceutical manufacturers are willing to produce children’s essential medicines, and just 0.2% of pharmaceutical enterprises are specialized in producing children’s medicines (Sun et al., 2018). Moreover, the centralized bidding procurement and distribution system for medicines had been gradually established in China (Wang and Ge, 2009). In 2018, to improve the drug procurement and pricing mechanisms, China conducted a pilot program of the centralized drug procurement in 11 cities. In 2019, the volume-based purchasing pilot program, using a competitive bidding process to purchase high-quality generic drugs for which branded drug substitutes were available, further expanded the implementation. The objective of volume-based purchasing policy was to allow substitution of branded drug products with generic drug products, which was intended to promote cost savings. Therefore, the availability of OBs medicines could be affected. And the National Health Commission of China included the use proportion of volume-based purchasing drugs in the performance assessment of tertiary public medical institutions, which might lead tertiary public medical institutions to give priority to use generic drug products. Therefore, the reasons might explain the poor availability of medicines for children, especially for OBs. Usually, only the pharmaceutical firms that offer the lowest prices could win bids, which probably leads to shrinking revenues from medicine sales. To win the bid, some pharmaceutical companies have to decrease the medicine price (Jiao, 2013), but there are few price controls on active pharmaceutical ingredients (APIs). The high cost of APIs and low profits are the important reason for the shortage of medicines. In 2018, the “Early warning of medicines that are easy to be shortage” of the Liaoning Province in China, reported that 81 medicines could not be supplied in time, and the main reason was the increase in the price of APIs. Moreover, the cross-regional centralized drug procurement expanded the scope of the suppliers’ distribution area, which is undoubtedly a greater challenge for distributors, and it is another important reason (Liu and Mao, 2021). Besides, in public sector, inadequate funding, lack of incentives for maintaining stocks, and inability to forecast clinical needs accurately (Cameron et al., 2009) could be also the reasons for the low availability of medicines for children.

Compared with other previous studies (Wang et al., 2017; Sun et al., 2018), a slightly different result of this study was showed that the median of MPRs for LPGs was 4.28, which meant the price of essential medicine for children might be unreasonable in public sector. The results might be related to the selected survey medicines, because we found the median of MPRs for injection was 4.32 and was much higher than tablet/capsule whose median of MPRs was 0.68. Compared with the findings of the adult’s medicine price survey that had been conducted in Sichuan Province (Yang et al., 2015), the price of children’s LPGs seemed to be higher than adult’s medicines. In general, the prices of essential medicines for children were higher than IPRs, especially for many injections.

We evaluated the affordability of medicines for six common childhood conditions. Most LPGs were affordable in public sector, namely, the standard treatments cost less than 1 day’s wage. This finding was similar to the previous study in China (Wang et al., 2017; Sun et al., 2018). But our study found ceftriaxone injection and cefazolin injection treatment cost over 10 days’ wage and were unaffordable, no matter in urinary tract infection, or community-acquired pneumonia standard treatments. This might be related to the higher unit price of these injections.

Relevant measures should be taken to improve access to medicines for children. From a policy perspective, learn from other countries’ experience, and formulate relevant policies and suggestions to guarantee the accessibility of medicines for children in China. And the government should develop a list of national essential medicines for children, and take some measures and make some policies to mobilize pharmaceutical companies to produce children’s medicines. Moreover, investigate the pediatrician’s knowledge and use of essential medicines, to promote the use of essential medicines for children. In addition, to reduce the burden of children’s diseases, it is recommended that medicare should include more children’s medicines.

## Conclusion

Although the availability of LPGs for children was higher than OBs in public sector, the availability of children’s essential medicines was still low in surveyed public sector in Sichuan Province, which was similar to previous studies in other provinces of China. The price of most medicines surveyed was higher than their IRPs in public sector, especially for some injections. The affordability of most surveyed LPGs was reasonable in public sector, except ceftriaxone injection and cefazolin injection.

## Data Availability

The original contributions presented in the study are included in the article/Supplementary Material, further inquiries can be directed to the corresponding authors.
